# Lactate Upregulates the Expression of DNA Repair Genes, Causing Intrinsic Resistance of Cancer Cells to Cisplatin

**DOI:** 10.3389/pore.2021.1609951

**Published:** 2021-12-20

**Authors:** Marzia Govoni, Valentina Rossi, Giuseppina Di Stefano, Marcella Manerba

**Affiliations:** Department of Experimental, Diagnostic and Specialty Medicine (DIMES), University of Bologna, Bologna, Italy

**Keywords:** DNA repair, cisplatin, glycolysis, lactate, DNA damage

## Abstract

Intrinsic or acquired drug resistance is one of the major problems compromising the success of antineoplastic treatments. Several evidences correlated some therapeutic failures with changes in cell metabolic asset and in line with these findings, hindering the glycolytic metabolism of cancer cells via lactate dehydrogenase (LDH) inhibition was found to overcome the resistance to chemotherapeutic agents. Lactate, the product of LDH reaction, was shown to be involved in epigenetic regulation of gene expression. The experiments described in this paper were aimed at highlighting a possible direct effect of lactate in modifying the response of cancer cells to a chemotherapeutic treatment. To discriminate between the effects potentially caused by glycolytic metabolism from those directly referable to lactate, we selected cancer cell lines able to grow in glucose deprived conditions and evaluated the impact of lactate on the cellular response to cisplatin-induced DNA damage. In lactate-exposed cells we observed a reduced efficacy of cisplatin, which was associated with reduced signatures of DNA damage, enhanced DNA recombination competence and increased expression of a panel of genes involved in DNA repair. The identified genes take part in mismatch and nucleotide excision repair pathways, which were found to contribute in restoring the cisplatin-induced DNA damage. The obtained results suggest that this metabolite could play a role in reducing the efficacy of antineoplastic treatments.

## Introduction

The activated glucose metabolism of cancer cells is functional in coping with their increased energy demand and need of metabolic intermediates, required to build-up new macromolecules [1,2].

Although the discovery of this metabolic feature dates back to almost one century ago [3], it is only in recent years that evidences showing a direct correlation between enhanced glycolysis and changes in gene expression have been obtained. Indeed, the metabolic reprogramming of cancer cells was found to impact on distinct morphological features of cancer cell nucleus [4]. Metabolites originating during the glycolytic cascade have been shown to increase histone acetylation and promote an open chromatin structure [4,5], which facilitates the transcriptional and replication machineries triggered by oncogenes activation.

Interestingly, elevation of glycolysis seems to confer cancer cells resistance to ionizing radiation [6], while its inhibition results in compromised DNA repair [7].

Predictably, facilitated DNA repair could also impact on the response of cancer cells to chemotherapeutic agents, as suggested by several evidences correlating therapeutic failures with changes in cell metabolic asset [8].

Lactate dehydrogenase (LDH) activity is a nodal point for the maintenance of the glycolytic flux of cancer cells [9]. By reducing pyruvate to lactate, LDH rapidly restores NAD+, which is needed for the first steps of glucose metabolism. This enzyme is considered an interesting therapeutic target for developing new antineoplastic treatments and accumulating evidences show that its inhibition or reduced expression can be successful in increasing the efficacy of chemotherapeutic agents [10–13]. A possible explanation to these results resides in the block of energy metabolism potentially caused by LDH inhibition, which hinders the highly ATP consuming reactions involved in DNA repair. A further mechanism could be linked to the non-metabolic functions of this enzyme; in fact, the A isoform of LDH (LDH-A) was found to be located also in cell nucleus, where it takes part in transcription complexes regulating gene expression [14]. Finally, lactate (the product of LDH reaction) is one of the metabolic intermediates shown to be involved in the epigenetic modulation of gene expression [15,16] and for this reason it was proposed as an “oncometabolite” and a key mediator in the metabolic cross-talk between cancer cells and their microenvironment [17–19]. Similar to other metabolites, lactate can hinder HDAC function; furthermore, a possible histone modification through the “lactylation” of lysine residues has been documented [20]. This change was found to serve as an epigenetic modification that directly stimulates gene transcription from chromatin. Furthermore, it was shown to compromise doxorubicin antineoplastic effect [21–22].

With the experiments described in this paper, we explored a possible direct role of lactate in reducing the response of cancer cells to a chemotherapeutic treatment. To this aim, we also verified the effect of this metabolite on the expression of a panel of genes involved in DNA repair, predicting a functional interaction network between the proteins encoded by the upregulated genes. We used cultured human cancer cells maintained in conditions allowing to highlight a possible direct effect of lactate, ruling out interferences from other glycolytic intermediates.

## Materials and Methods

### Cell Cultures and Treatments

SW620 and HepG2 cells (ECACC, #87051203 and #85011430) were cultured in L-15 medium supplemented with 100 U/ml penicillin/streptomycin, 4 mM glutamine and 10% dialyzed FBS. This medium does not contain glucose. For some experiments, cells were also maintained in low-glucose DMEM, with standard supplementations. All the materials used for cell culture and all the reagents were obtained from Sigma-Aldrich, unless otherwise specified. Lactate (L-isomer) was always used at a 10 mM concentration and was administered in L-15 medium 48–72 h before experiments. Both cell cultures were found to express the MCT1 carrier for lactate uptake [23,24]. No significant reduction of lactate concentration in culture medium was observed up to 72 h (Supplementary Figure S1).

Cultures were routinely tested for Mycoplasma contamination and found to be free.

### Cell Viability Experiments

The effect of cisplatin (CPL, 0–50 μM) on cell viability was assessed at 24 h, in cultures maintained in L-15 medium with or without 10 mM lactate. Results were evaluated with the neutral red assay (NR), which allows a precise estimate of cell number [25]. Before each experiment, a plot reporting the NR absorbance values of scalar amounts of cells was obtained. These data were fitted by using the linear regression analysis; the resulting mathematical equation was used to calculate the number of cells at the end of experiments. SW620 and HepG2 cells (1.0 × 10^4^/well) were seeded in 96-multiwell plates. After 24 h treatment with CPL, they were maintained 3 h at 37°C with the NR dye, dissolved in medium at the final concentration of 30 μg/ml. Medium was then removed and the cells were solubilized with 200 μl of 1% acetic acid in 50% ethanol. Absorbance of the solutions was measured at λ540.

### Evaluation of Abasic DNA Sites

The amount of abasic (AB) sites on DNA after CPL treatment was evaluated using a commercially available assay from Cell Biolabs. This assay is based on the use of a probe (ARP) which specifically reacts with the aldehyde group on the open ring form of AB sites [26].

SW620 cells maintained in L-15 with or without 10 mM lactate were exposed for 90 min to CPL (0–50 μM). Genomic DNA was isolated using the phenol/chloroform/isoamyl alcohol extraction procedure [27]. The recovered, water-soluble material was then treated with 2 µg RNase (Thermo-Fisher Scientific) for 75 min at room temperature, after which the enzyme was removed by an additional step of phenol extraction. Finally, DNA was purified by ethanol precipitation. It was dissolved in a 10 mM Tris buffer, pH 7.5, containing 1 mM EDTA at a concentration of 100 μg/ml. Reaction with ARP was performed following the instructions of the assay’s manufacturer and the quantification of AB sites in the experimental samples was obtained by generating a standard curve using an ARP-DNA reference sample included in the assay. Experiment was repeated twice, with duplicate samples.

### Immunoblotting Experiments

These experiments were performed in SW620 and HepG2 cells cultured in L-15 with or without 10 mM lactate; immunoblotting was used to assess the level of H3 acetylation, H2AX phosphorylation (γ-H2AX, a marker of DNA damage [28]), TP73 and GSTP1. To assess DNA damage, cells were exposed to 12.5 μM CPL for 1 h; medium was then removed and cultures were maintained for additional 16, 24 and 40 h γ-H2AX level was evaluated at the end of each time interval.

For immunoblotting, cells (9 × 10^5^ in T25 flasks) were harvested and lysed in 60 µl RIPA buffer containing protease and phosphatase inhibitors. Proteins (30–50 µg) were loaded onto 4–12% precast polyacrylamide gel for electrophoresis and run at 170 V. Gels were blotted on a low fluorescent PVDF membrane (GE Healthcare) using a standard apparatus for wet transfer. The blotted membrane was blocked with 5% BSA in TBS-TWEEN and probed with the primary antibodies: rabbit anti-H3 (Cell Signaling); rabbit anti-Panacetyl-H3 (Active Motif), rabbit anti-γ-H2AX (phospho-S139) (Abcam); rabbit anti-TP73 and anti-GSTP1 (Thermo-Fisher Scientific); rabbit anti-β-actin, (Sigma-Aldrich). Binding was revealed by a Cy5-labelled secondary antibody (goat anti rabbit-IgG, GE Healthcare). Fluorescence of the blots was assayed with the Pharos FX Scanner (Bio-Rad) at a resolution of 100 µm.

### Study of Episomal Plasmid Recombination

The rate of episomal plasmid recombination in SW620 and HepG2 cells maintained in L-15 with or without 10 mM lactate was assessed by using a commercially available kit (Norgen Biotek). This assay is based on cell transfection with two plasmids that recombinate upon entry. Recombination efficiency can be assessed by real-time PCR, using the primer mixtures included in the assay kit, which allow to discriminate between the original plasmid backbones and their recombination product.

Cells were seeded in a 24-well plate (2 × 10^5^ cells/well, in duplicate) and allowed to adhere overnight. Co-transfection with the two plasmids was performed in Lipofectamine 2000 (Thermo-Fisher Scientific) for 5 h at 37°C. At the end of incubation, cells were washed with PBS and harvested; DNA was isolated using the QIAamp DNA mini kit (Qiagen). 25 ng of purified DNA was used for the real-time PCR, which was performed according to the protocol indicated by the manufacturer. Data analysis was based on the ΔΔCt method and compared the level of recombination assessed in lactate-exposed cells to that measured in control cultures.

### Real-Time PCR Array of DNA Repair Genes

This experiment was performed on SW620 and HepG2 cultures, maintained in L-15 with or without 10 mM lactate (72 h). For comparison, a similar experiment was also performed on cultures grown in DMEM (a medium allowing glycolytic metabolism), exposed for 16 h to 40–80 mM oxamate (OXA), a LDH inhibitor hindering glucose metabolism [29].

RNA was extracted from exponentially growing cells seeded in T75 flasks, using the method described in [30]. RNA quantity and quality were assessed spectrophotometrically; for each sample, 2 μg RNA was retro-transcribed with the iScript gDNA Clear cDNA Synthesis kit (Bio-Rad). The expression of DNA damage and repair genes was analyzed using the DNA Damage Tier 1 H96 PrimePCR™ Assay (Bio-Rad). Real-time PCR was conducted as indicated by the manufacturer, in a CFX96 real-time cycler (Bio-Rad). The validation data for this array are available online at: https://www.bio-rad.com/en-uk/prime-pcr-assays/predesigned-plate/sybr-green-dna-damage-tier-1-h96.

The same experimental conditions were followed also for the additional PCR assays reported in Figure 4D. The primers’ sequences used for assessing the expression of the genes reported in Figure 4D, as well as those used for the internal control genes, are reported in the Supplementary Material (Supplementary Table S1).

### Statistical Analyses

All data were analyzed by using the GraphPad Prism software. Results were obtained from at least two independent experiments. They are expressed as mean ± SE of replicate values; the significance level was set at *p* < 0.05.

## Results

### Lactate-Exposed SW620 Cells Showed Reduced Response to Cisplatin

CPL was selected as a representative chemotherapeutic agent for the experiments since the pattern of DNA damage produced by this drug has been extensively studied. CPL appeared to potentially trigger all the principal DNA repair pathways: nucleotide excision repair, mismatch repair, homologous recombination and non-homologous end joining [31].

Reduced response to chemotherapeutic agents has been often correlated with increased glycolytic metabolism [8]. In order to evidence a direct contribute of lactate in this phenomenon, we searched human cancer cell lines able to grow in glucose deprived conditions (L-15 medium). According to the ATCC and ECACC indications, L-15 is the optimal medium for culturing the SW620 colon adenocarcinoma cells and, for this reason, they were used in all the reported experiments.

**FIGURE 1:**
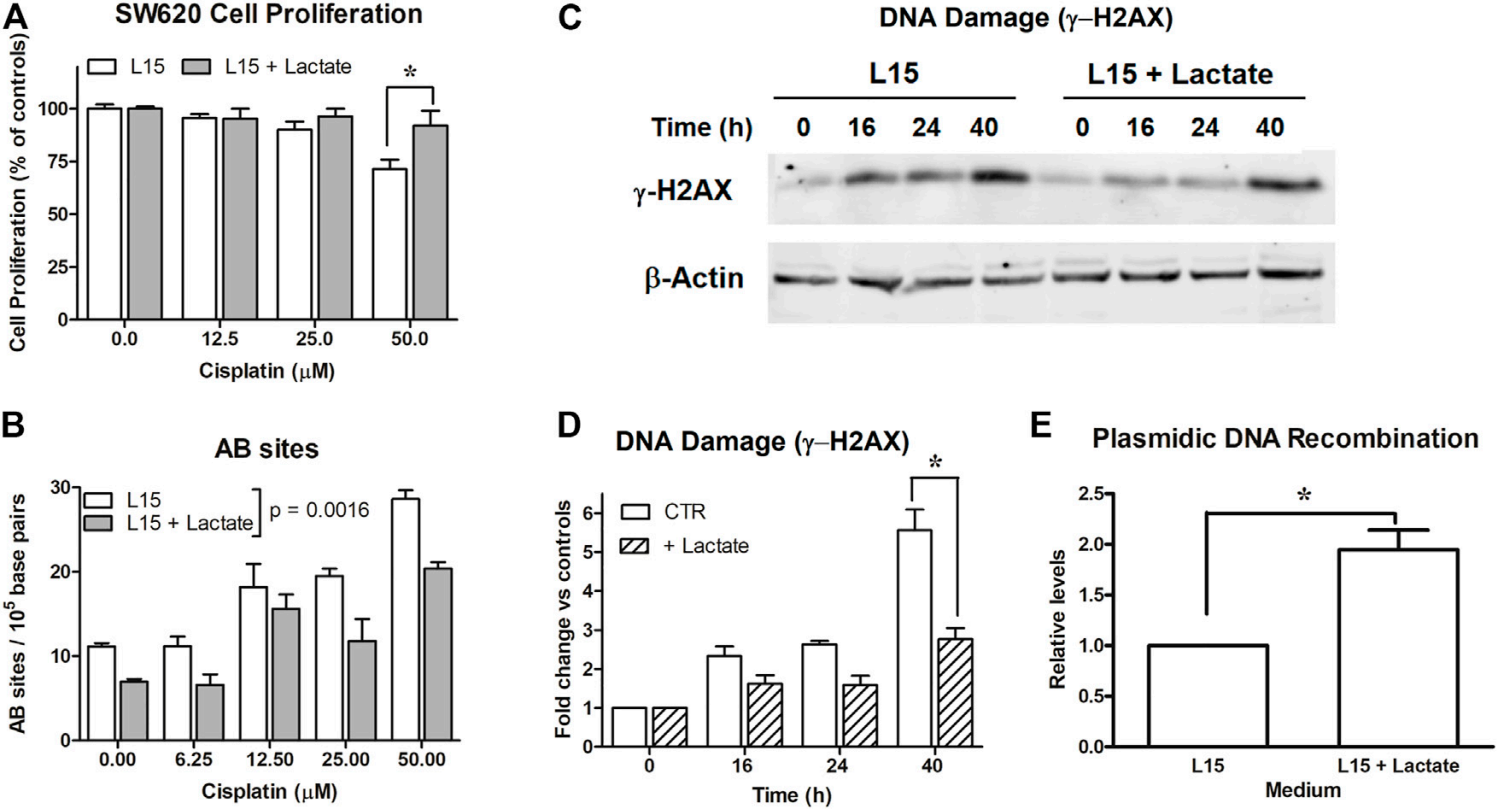
Experiments on SW620 cells. **(A)**: Antiproliferative effects caused by cisplatin on SW620 cultures maintained in L-15. *, *p* < 0.05. **(B)**: Evaluation of AB sites in cultures exposed to scalar doses of cisplatin (0–50 μM) for 90 min, with or without 10 mM lactate. Lactate in medium was found to significantly reduce AB sites (*p* = 0.001). **(C)**: Evaluation of DNA damage (γ-H2AX) in cells maintained with or without 10 mM lactate and exposed to cisplatin (12.5 μM). The densitometric reading of band intensities is shown in **(D)**. *, *p* < 0.001, compared to control cultures. **(E)**: DNA recombination competence assessed in cultures exposed to lactate. *, *p* < 0.05, compared to control cultures.

SW620 cells were maintained in L-15 and probed with 0–50 μM CPL for 24 h, with or without a supplementation of 10 mM lactate. This dose of lactate was chosen on the basis of previously published works, suggesting that in cancer cells and extracellular milieu the concentration of this metabolite easily reaches or even overcomes this level [16]. The obtained results are reported in Figure 1A. Data were analyzed by two-way ANOVA; according to Bonferroni’s post-test, lactate in medium significantly reduced the antiproliferative effect of 50 μM CPL (*p* < 0.05).

### The Reduced Response to Cisplatin Caused by Lactate was Associated With Decreased Signatures of DNA Damage and Upregulated DNA Recombination Competence

To explain the data of Figure 1A, we estimated the extent of DNA damage caused by CPL in SW620 cells grown in L-15, with or without 10 mM lactate. In the first experiment, DNA damage was evaluated by quantifying the presence of AB sites [26]. For this assay, cells were exposed for 90 min to 0–50 μM CPL. Results are shown in Figure 1B; they were statistically analyzed using two-way ANOVA. Lactate in medium was found to significantly decrease the number of AB sites, with *p* = 0.0016.

This experiment showed that initial evidences of DNA damage are obtained starting from 12.5 μM CPL; for this reason, this dose was also applied for assessing γ-H2AX levels [28] (Figures 1C,D). In the different samples, the γ-H2AX band intensity was normalized on β-actin; the results of the densitometric reading are reported in the bar graph (Figure 1D). Data were analyzed by two-way ANOVA; Bonferroni’s post-test indicated a significantly reduced γ-H2AX level in lactate-exposed cells at the 40-h time interval (*p* < 0.001).

Taken together, these findings suggested enhanced competence in managing DNA damage in cells exposed to lactate. Interestingly, these cells also displayed improved DNA recombination, which was observed independently of CPL exposure.

SW620 cultures were transfected with a couple of plasmids reproducing the LacZ sequence as a result of their recombination. This sequence can be detected by real-time PCR. As shown in Figure 1E, lactate-exposed SW620 cells revealed an almost doubled capacity of generating the LacZ sequence, which suggests enhanced activity of enzymes involved in the DNA recombination process. The data of Figure 1E were obtained from three independent experiments and were analyzed by applying the paired t-test, which compared the increase measured in lactate-exposed SW620 cultures to the recombination level measured in their respective control cultures, set to 1; *p* value was 0.0389.

### Real-Time PCR Array of DNA Repair Genes

To identify the DNA repair genes upregulated by lactate, we applied to SW620 cells a real-time PCR array specifically developed to study DNA damage and repair (Tier1 H-96 Prime PCR Array).

Experiment was repeated twice and the obtained results were processed with the aid of a dedicated software. The complete list of genes included in this array, together with the internal controls of the PCR reaction, is available at: https://www.bio-rad.com/en-uk/prime-pcr-assays/predesigned-plate/sybr-green-dna-damage-tier-1-h96. Moreover, a table reporting the extended names of the genes cited in the Results’ section and in figures have been included in the Supplementary Material file (Supplementary Table S2).

**FIGURE 2:**
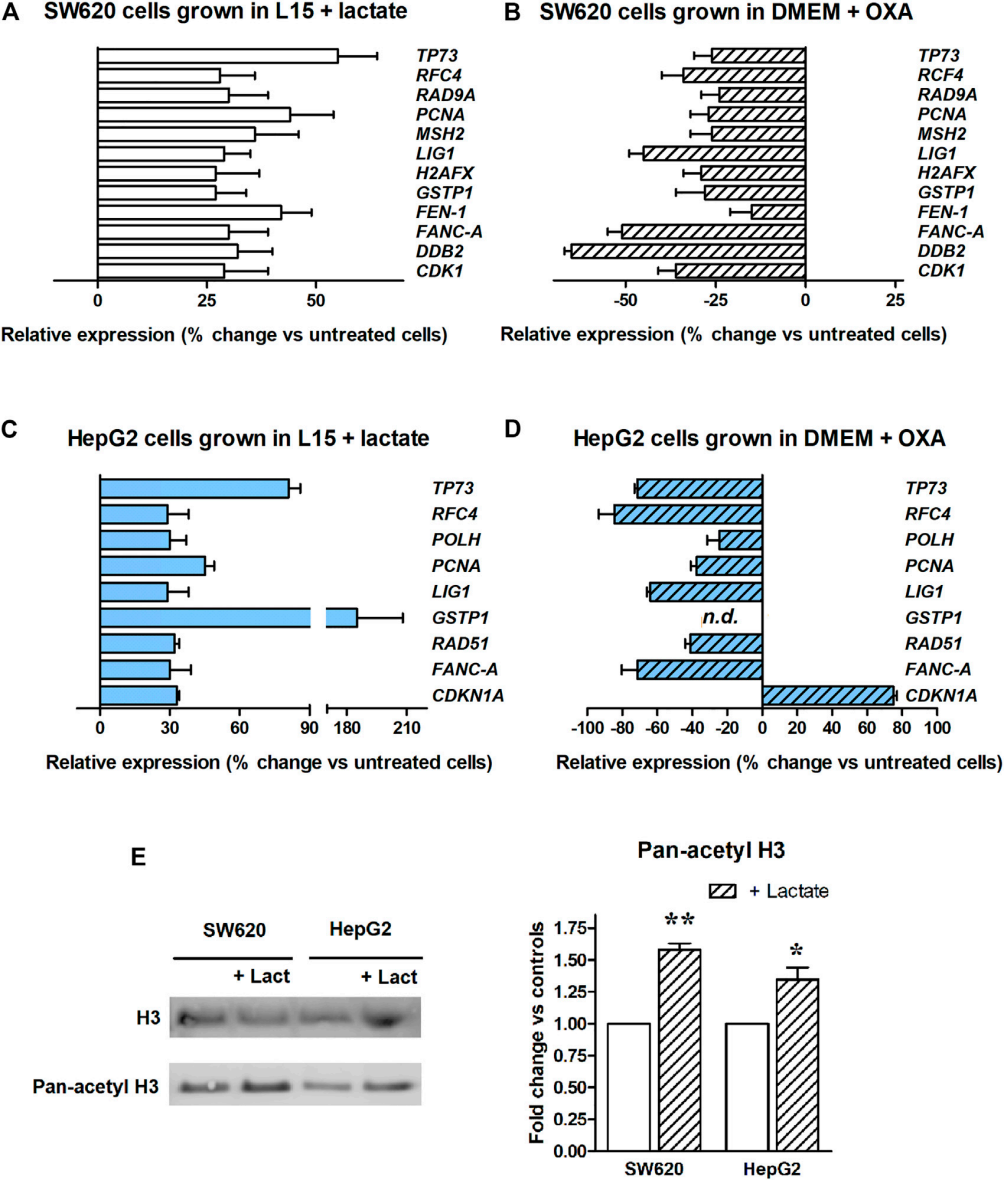
Real-time PCR array of DNA repair genes. The bar graphs display genes showing a >25%-increased expression. **(A)**: Experiment performed in SW620 cells maintained in L-15 medium and exposed to lactate. All the reported data were found to be statistically significant, with the exception of H2AFX and CDK1. **(B)**: Experiment performed in glycolyzing SW620 cells exposed to OXA. Lactate depletion caused by OXA significantly reduced the expression of all the genes, with the exception of FEN1. **(C)**: Experiment performed in HepG2 cultures maintained in L-15 medium and exposed to lactate. All the reported data were found to be statistically significant. The used statistical analysis and the obtained *p* values are reported in the text. **(D)** Experiment performed in glycolyzing HepG2 cells exposed to OXA. Lactate depletion caused by OXA significantly reduced the expression of all the genes, with the exception of CDK1NA. n.d.: GSTP1 expression was not detected in OXA exposed cells. **(E)** Level of H3 acetylation assessed in lactate-exposed cultures. * and **, *p* < 0.05 and <0.01, compared to control cultures, respectively.

In evaluating the obtained results, a lower threshold at 25%-increased expression was set, since comparable effects have been reported in previous studies examining the epigenetic effects of lactate in different experimental settings [22]. Among the 88 genes included in the array, 12 showed a >25% upregulation following lactate exposure; they are reported in the bar graph of Figure 2A. Results were analyzed using the column statistics’ function of the GraphPad software by applying the one-sample t-test, which computes whether the mean of each data set is different from a given hypothetical value (0, i.e., no change, compared to untreated cultures). All the reported data were found to be statistically significant with the exception of the genes for Cyclin Dependent Kinase 1 (CDK1) and for H2AX variant Histone (H2AFX). The statistically significant changes showed *p* values ranging from 0.045 to 0.009.

To confirm the findings of Figure 2A, additional PCR array experiments were performed on SW620 cells grown in DMEM. Contrary to L-15, DMEM contains glucose and allows the proceeding of glycolytic flux up to lactate. For these PCR experiments we exposed glycolyzing SW620 cells to 40 mM OXA for 16 h. OXA is a pyruvate analog which specifically inhibits LDH [29]; in preliminary experiments, we found that a 40 mM dose of this inhibitor almost completely prevents lactate production in glycolyzing SW620 cells, without reducing their ATP level and viability (Supplementary Material, Supplementary Figure S2). Figure 2B shows that when glycolyzing SW620 cells were exposed to OXA, all the 12 genes identified in the previous PCR array (Figure 2A) reduced their expression below the levels measured in untreated, glycolyzing cells. With the exception of Flap structure-specific Endonuclease 1 gene (*FEN1*), all the observed reductions were found to be statistically significant; *p* values ranged from 0.039 to <0.0001. Interestingly, in lactate-deprived cultures a statistically significant reduction was observed also for *CDK1* and *H2AFX*.

To extend our observations, we wondered whether the changes in gene expression caused by lactate could differ among cell types. For this reason, we searched a second culture able to grow in the same glucose-deprived condition as SW620 cells, but from a different tissue. The HepG2 hepatoma cell line was found to tolerate the glucose-deprived L-15 medium and was then used for additional PCR array experiments, aimed at evaluating the expression of DNA repair genes after lactate exposure. Results are shown in Figure 2C. In this case, we found a >25% upregulation in 9 genes and, interestingly, 6 of them were in common with SW620 cells. Results were analyzed as described for SW620 cells. In HepG2 cells, the observed upregulation reached the level of statistical significance for all genes; *p* values ranged from 0.048 to <0.0001. When these cells were maintained in DMEM and exposed to the OXA dose preventing lactate production (80 mM, Supplementary Figure S2), all the observed changes were reversed, except for *CDK1NA*, which was further increased. The *p* values ranged from 0.039 to <0.0001. The antiproliferative effect caused by OXA in DMEM-cultured HepG2 cells (see Supplementary Figure S2) could explain the finding concerning *CDK1NA* (a cell cycle regulator).

Figure 2E shows that lactate-exposed SW620 and HepG2 cells displayed a significantly increased level of H3 acetylation, suggesting inhibition of HDAC as the mechanism underlying the effects observed in Figures 2A,C.

### Experiments on Lactate-Exposed HepG2 Cells

**FIGURE 3:**
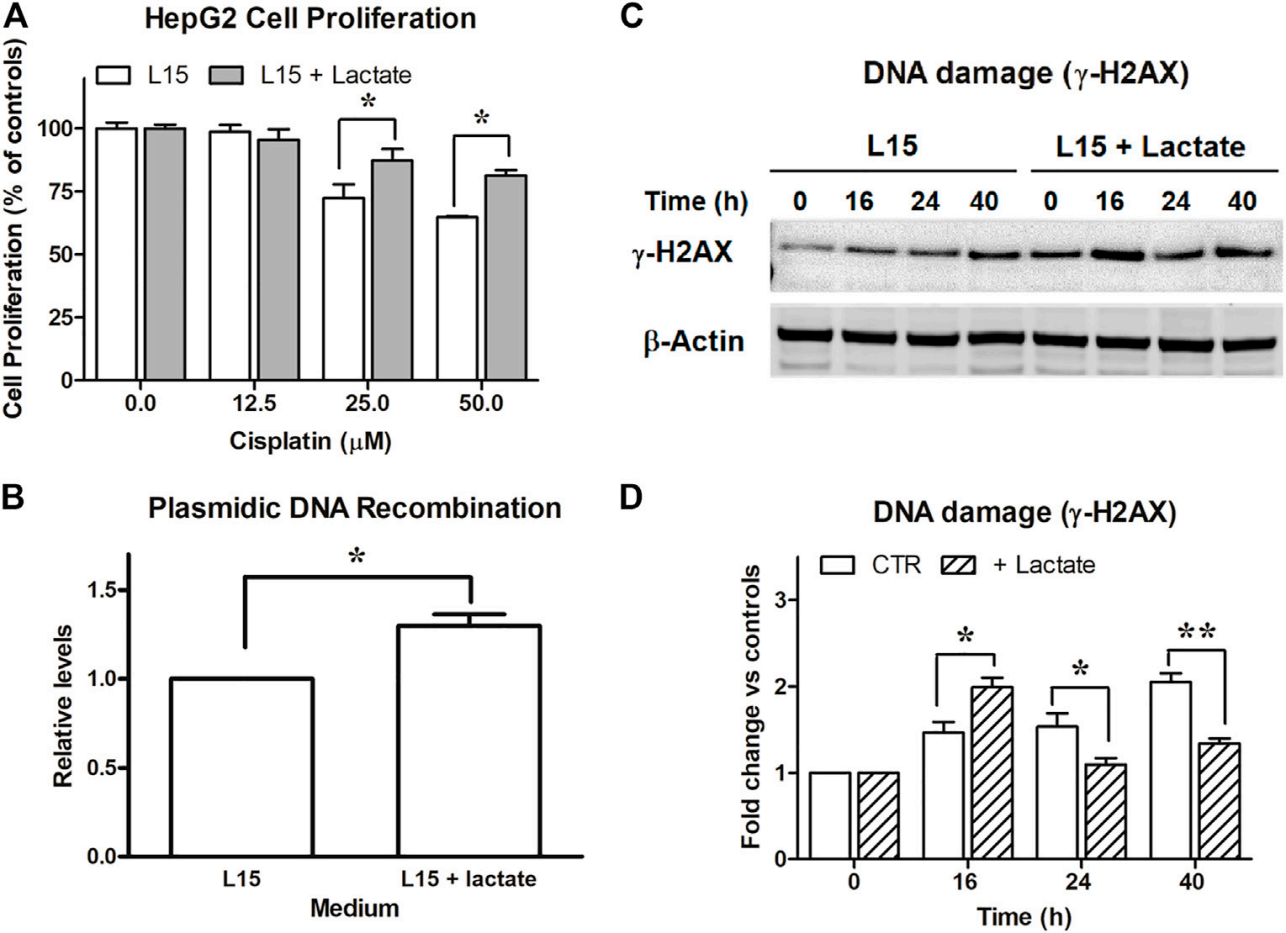
Experiments on HepG2 cells. **(A)**: Antiproliferative effects caused by cisplatin on HepG2 maintained in L-15 and exposed to 10 mM lactate. *, *p* < 0.05, compared to control cultures. **(B)**: DNA recombination competence, assessed as described for SW620 cells. *, *p* < 0.05. **(C)**: Evaluation of DNA damage following CPL exposure in cells maintained with or without 10 mM lactate and assessed by immunoblotting evaluation of γ-H2AX. The densitometric reading of band intensities is shown in **(D)**. Lactate-exposed cells showed a constitutively higher γ-H2AX signal which, after CPL treatment, peaked at T = 16 h. At later time intervals, the γ-H2AX signal increase referred to T = 0 (fold change) was significantly lower in lactate-exposed cells, when compared to control cultures. *, *p* < 0.05; **, *p* < 0.01.

Following these results, we also investigated whether a different susceptibility to DNA damage could also be detected in lactate-exposed HepG2 cultures, as observed for SW620 cells. Unfortunately, these experiments were hindered by the compromised proliferation shown by these cells in L-15; the obtained results are reported in Figure 3. The data of Figures 3A,B were in line with those previously observed in SW620 cells. They were statistically analyzed as described for the corresponding experiments in Figures 1A,E. In the experiment of Figure 3A, lactate was found to significantly reduce the efficacy of 25 and 50 μM CPL (*p* < 0.05, according to Bonferroni’s post-test). In the experiment of Figure 3B, the plasmidic DNA recombination detected in lactate-exposed cells was significantly increased (*p* = 0.045).

The study of γ-H2AX (Figure 3C) showed in HepG2 cells a DNA damage signaling pattern different from that observed in SW620 cultures (Figures 1C,D). A γ-H2AX level constantly increasing over time was observed in control cells. Lactate-exposed cultures showed a constitutively higher γ-H2AX signal, which after CPL treatment peaked at 16 h. However, when compared to T = 0, its further increase over time (fold change) was significantly lower than that measured in control cultures not exposed to lactate: at 40 h, a 2-fold increased signal was detected in control cultures, while a 1.3-fold increase was measured in lactate-exposed cells. Data of Figure 3C were statistically analyzed as described for the similar experiment performed on SW620 cells (Figure 1C). The increase of γ-H2AX signal (fold change) in lactate-exposed cultures was significantly lower at 24 and 40 h (*p* < 0.05 and 0.01, respectively, according to Bonferroni’s post-test).

### Functional Interaction Network of Upregulated Genes

The PCR array experiments allowed us to identify a cluster of 6 genes which in both SW620 and HepG2 cells appeared to be potentially regulated through the level of this metabolite: Proliferating Cell Nuclear Antigen (*PCNA*), Tumor Protein p73 (*TP73*), Replication Factor C subunit 4 (*RFC4*), Fanconi Anemia complementation group A (*FANC-A*), DNA Ligase 1 (*LIG1*), Glutathione S-Transferase π1 (*GSTP-1*). To identify the functional connections between the corresponding proteins, we used the Search Tool for the Retrieval of Interacting Genes (STRING) database, a resource which can be reached at: http://string-db.org. The STRING database is able to construct interaction networks among genes, also providing a confidence score; moreover, by applying the Kyoto Encyclopedia of Genes and Genomes (KEGG) analysis, it identifies their related biochemical pathways and cellular functions.

**FIGURE 4:**
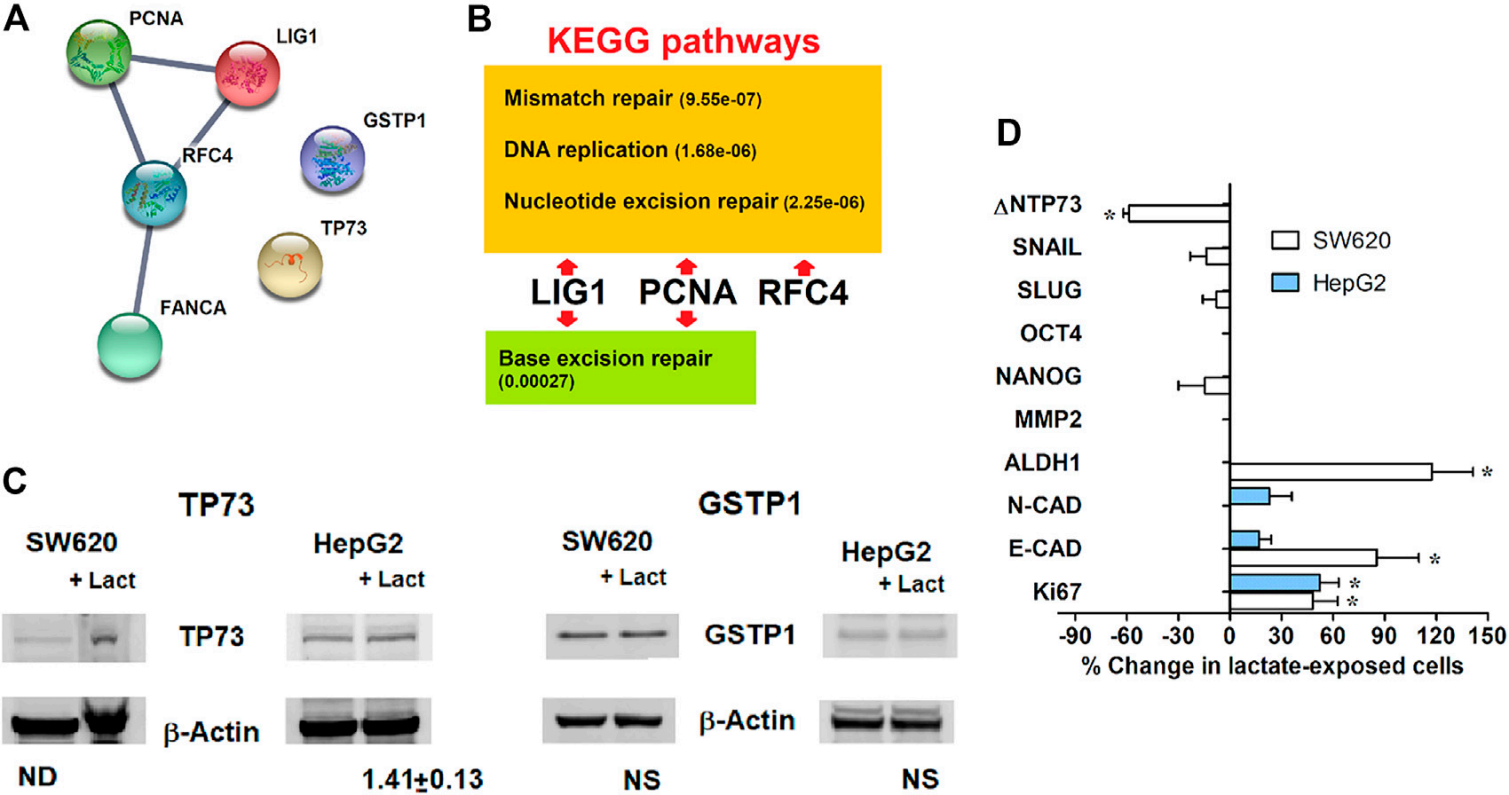
**(A)**: Functional interaction network among the genes identified using the real-time PCR array; downloaded from http://string-db.org. The edge thickness between protein nodes is indicative of a 0.9 confidence score. **(B)**: Biochemical pathways identified by the KEGG analysis. In parentheses, false discovery rates. **(C)**: Immunoblotting evaluation of the proteins not included in the identified DNA repair pathways. The level of TP73 was found to be increased in both lactate-exposed cultures. **(D)**: Expression of representative genes associated with proliferative potential and stem properties, assessed in lactate-exposed SW620 and HepG2 cells. Data were analyzed using the column statistics function of the Prism software, as described for the PCR array of DNA repair genes; *, statistically significant changes; *p* values ranged from 0.040 to 0.004. Missing bars in the graph denote undetectable mRNA.

Figures 4A,B show the obtained results. In building the interaction network, the edges representing gene-gene associations have been set on the highest confidence interaction score (0.9), to increase the strength of data support. This setting resulted in the identification of a functional network involving four of the analyzed genes, which gave an interaction enrichment *p* value = 7.35 × 10^−05^. Accordingly, three of the four identified KEGG pathways showed very low false discovery rates, reported in the scheme of Figure 4B. All of them concern the interaction between *LIG1*, *PCNA* and *RFC4*. Interestingly, the identified pathways include mismatch and nucleotide excision DNA repair, which were found to be involved in cellular response to CPL damage [31]. These data can give a mechanistic explanation to the results obtained in lactate-exposed SW620 and HepG2 cells, treated with CPL; together with the data of Figures 1–3, they suggest that the increased gene expression caused by lactate can result in enhanced protein function, leading to modified cell response to DNA damaging agents. According to Figure 4A, *TP3* and *GSTP1* cannot be included in the gene network involved in the response to CPL. For this reason, we analyzed the level of the corresponding proteins by immunoblotting. Results (Figure 4C) showed increased level of TP73 in both lactate-exposed cultures, while GSTP1 protein appeared to be unchanged. GSTP1 belongs to the family of phase II detoxification enzymes, the activity of which is commonly induced by exposition to xenobiotics [32]; for this reason, it can be hypothesized that the upregulated *GSTP1* gene expression caused by lactate is not sufficient for obtaining enhanced protein levels.

Finally, we focused our attention on the two genes showing the highest increased expression in lactate-exposed cells: *PCNA* and *TP73* (Figures 2A,C). PCNA is a DNA polymerase accessory factor playing a regulatory role in both DNA repair and replication [33,34]. It was found to be preferentially expressed in actively proliferating human cancer cells and in transformed normal cells; moreover, it has also been widely used as a tumor marker. TP73 is a member of the TP53 family showing prognostic significance [35]. *TP73* can be translated into different isoforms with opposite functions; in particular, the A isoform (TAp73) shows tumor-suppressor activity, while the Dominant-Negative isoform (ΔNTP73) fails to induce apoptosis and cell cycle arrest. It negatively regulates TP53 and TAp73 by acting as negative dominant. The primer sequences used for the DNA-damage PCR assay did not allow to discriminate between the TP73 isoforms.

For these reasons, an additional PCR study was performed to better characterize the phenotypic changes induced in both SW620 and HepG2 cells by lactate exposure. We analyzed the expression of representative genes associated with proliferative potential and stem cell properties. Results are shown in Figure 4D.

Unfortunately, in HepG2 cultures the only detectable genes were those of the epithelial and neural cadherins (*E-* and *N-CAD*) [36] and *Ki67* [37]. No significant changes were observed concerning *E-* and *N-CAD*; a significant increase was detected for *Ki67* expression (*p* < 0.05). An upregulation of this proliferation marker was also found in SW620 cells; in both cell lines, the increased expression of *Ki67* fits well with the data of PCNA expression.

In SW620 cells, lactate was found to markedly increase *E-CAD* levels and to cause reduced ΔNTP73. Together with the unchanged levels of the colon cancer stem markers *NANOG*, *SLUG* and *SNAIL* [38], these effects suggest that the increased trend in cell proliferation usually associated with Ki67 and PCNA is not characterized by phenotypic traits suggesting cancer progression, at least in this cell model. This idea is in line with the markedly upregulated expression of *GSTP1*, primarily observed in HepG2 cells (Figure 2C); the clinical significance of this parameter was repeatedly investigated in hepatocellular carcinoma and was found to correlate with a favorable prognosis [39].

Notably, in lactate-exposed SW620 cultures a doubled level of Aldehyde Dehydrogenase (*ALDH1*) was observed, suggesting that the increased resistance of these cells to CPL could also be linked to their higher capacity to cope with oxidative stress [40].

## Discussion

A possible role of lactate in regulating gene expression was hypothesized about 20 years ago [41]. Recently, new data have obtained, suggesting for lactate an important role in linking the metabolic state of the cell to gene expression [15–17].

Lactate has been defined a “mirror and motor” of tumor malignancy [42–44], since the metabolic program characterized by increased glycolysis and lactate production supports neoplastic change and tumor progression. Increased glycolysis is also associated with one of the most serious problems complicating cancer treatment, the increased drug resistance [8].

A better understanding of the linkages between the drug resistant phenotype and glycolytic metabolism is complicated by the difficulty of discriminating between a possible effect of lactate from that of other intermediates originating from glucose metabolism, for which a role in transcriptional regulation has also been suggested [4,5].

The experiments described here attempted to address this issue. To our knowledge, our experiments showed for the first time a direct effect of this metabolite on the expression of genes needed for mismatch and nucleotide excision DNA repair, which appeared to compromise the antineoplastic efficacy of cisplatin. Our data suggest that the increased lactate production of cancer cells could facilitate the onset of chemotherapy resistance.

## Data Availability

The original contributions presented in the study are included in the article/Supplementary Material, further inquiries can be directed to the corresponding author.
