# Supplementary material for: Lactate Upregulates the Expression of DNA Repair Genes, Causing Intrinsic Resistance of Cancer Cells to Cisplatin
Source: Pathol Oncol Res. 2021 Dec 20;27:1609951. doi: 10.3389/pore.2021.1609951 (PMC8720744; doi:10.3389/pore.2021.1609951)
Supplement: Supplementary file 1 [file DataSheet1.PDF]

Supplementary Material

Table S1
List of primer sequences used for the RT-PCR analysis reported in Table 1.

Gene	Forward primer (5' – 3')	Reverse primer (5' – 3')
Ki67	GAGGTGTGCAGAAAATCCAAA	CTGTCCCTATGACTTCTGGTTGT
E-CAD	CCCGGGACAACGTTTATTAC	GCTGGCTCAAGTCAAAGTCC
N-CAD	GGTGGAGGAGAAGAAGACCAG	GGCATCAGGCTCCACAGT
ALDH1	TGGATCAACTGCTACAACGC	CACTTCTGTGTATTCGGCCA
MMP2	TCACTCCTGAGATCTGCAAACAG	TCACAGTCCGCCAAATGAAC
NANOG	TTTGTGGGCCTGAAGAAAACT	AGGGCTGTCCTGAATAAGCAG
OCT4	CGACCATCTGCCGCTTTGAG	CCCCCTGTCCCCCATTCCTA
SLUG	TCGGACCCACACATTACC	CTACACAGCAGCCAGATTC
SNAIL	GCACATCCGAAGCCACAC	AGAAGGTCCGAGCACACG
ΔNP73	CAAACGGCCCGCATGTTCCC	TTGAACTGGGCCGTGGCGAG
β_2M	CATTCCTGAAGCTGACAGCATTC	TGCTGGATGACGTGAGTA
HPRT1	AGACTTTGCTTTCCTTGGTCAGG	GTCTGGCTTATATCCAACACTTCG
RPLP0	CAGATTGGCTACCCAACTGTT	GGCCAGGACTCGTTTGTACC
TUB-A	TGGAACCCACAGTCATTGATGA	TGATCTCCTTGCCAATGGTGTA

Notes:
β_2M and HPRT1 were used as internal controls in the experiments on HepG2 cells
RPLP0 e TUBA were used as internal controls in the experiments on SW620 cells

Table S2
Extended denominations of genes cited in abbreviated form in the Results' section and in Figures

	**Extended name**		**Extended name**
ALDH1	Aldehyde Dehydrogenase 1	*NANOG*	Homeobox protein Nanog
CDK1	Cyclin Dependent Kinase 1	*N-CAD*	Neural Cadherin
CDKN1A	Cyclin Dependent Kinase inhibitor 1A	*OCT4*	Octamer-binding transcription factor 4
DDB2	Damage specific DNA Binding protein 2	*PCNA*	Proliferating Cell Nuclear Antigen
E-CAD	Epithelial Cadherin	*POLH*	DNA Polymerase Eta
FANC-A	Fanconi Anemia complementation group A	*RAD51*	RAD51 Recombinase
FEN1	Flap structure-specific Endonuclease 1	*RAD9A*	RAD9 checkpoint clamp component A
GSTP1	Glutathione S-Transferase π1	*RFC4*	Replication Factor C subunit 4
H2AFX	Gene for H2A X variant Histone	*SLUG*	Snail family transcriptional repressor 2
H2AX	H2A X variant Histone	*SNAIL*	Zinc finger protein SNAI1
Ki67	Marker of proliferation Ki-67	*STRING*	Search Tool for the Retrieval of Interacting Genes
LIG1	DNA Ligase 1	*TAP73*	Isoform of TP73
MMP2	Matrix Metallopeptidase 2	*TP73*	Tumor Protein p73
MSH2	MutS Homolog 2	ΔNTP73	Dominant-Negative isoform of TP73

Figure S1

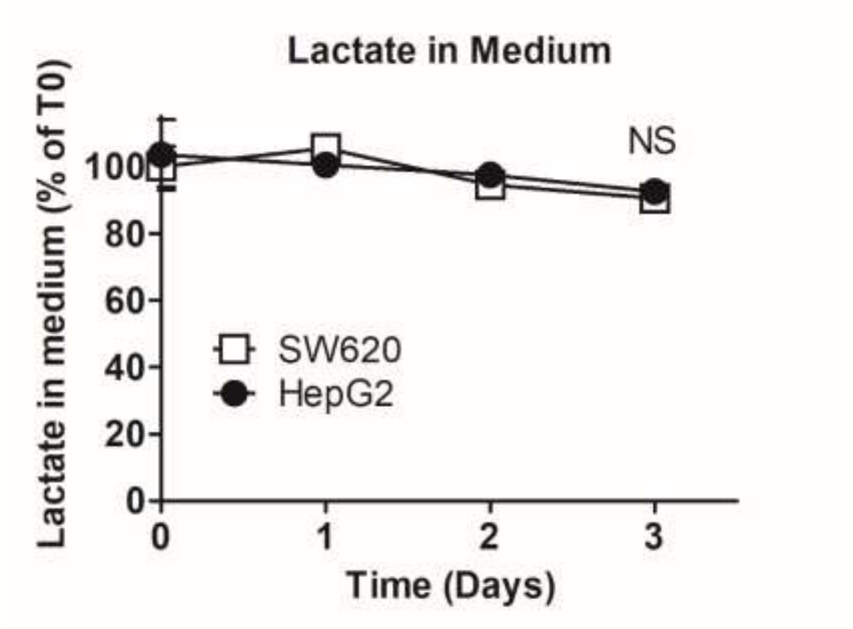

Evaluation of lactate concentration in L15 medium during cell culture. To evaluate the stability of lactate concentration in culture medium during the progression of experimental procedures, SW620 and HepG2 cells were maintained in T25 flasks and exposed to 5 ml of L15 medium supplemented with 10 mM lactate. At the times intervals indicated in the figure, 1 ml medium was collected and treated with 100 µl 100% TCA to remove serum proteins. Lactate in the acid soluble fraction was assessed by using the colorimetric method of Barker and Summerson and following the procedure described by Farabegoli et al. (Eur. J. Pharm. Sci. 2012; 47: 729–738). Data were statistically analyzed by one-way ANOVA; no statistically significant decrease in lactate level was observed with the 3 days of culture.

Figure S2

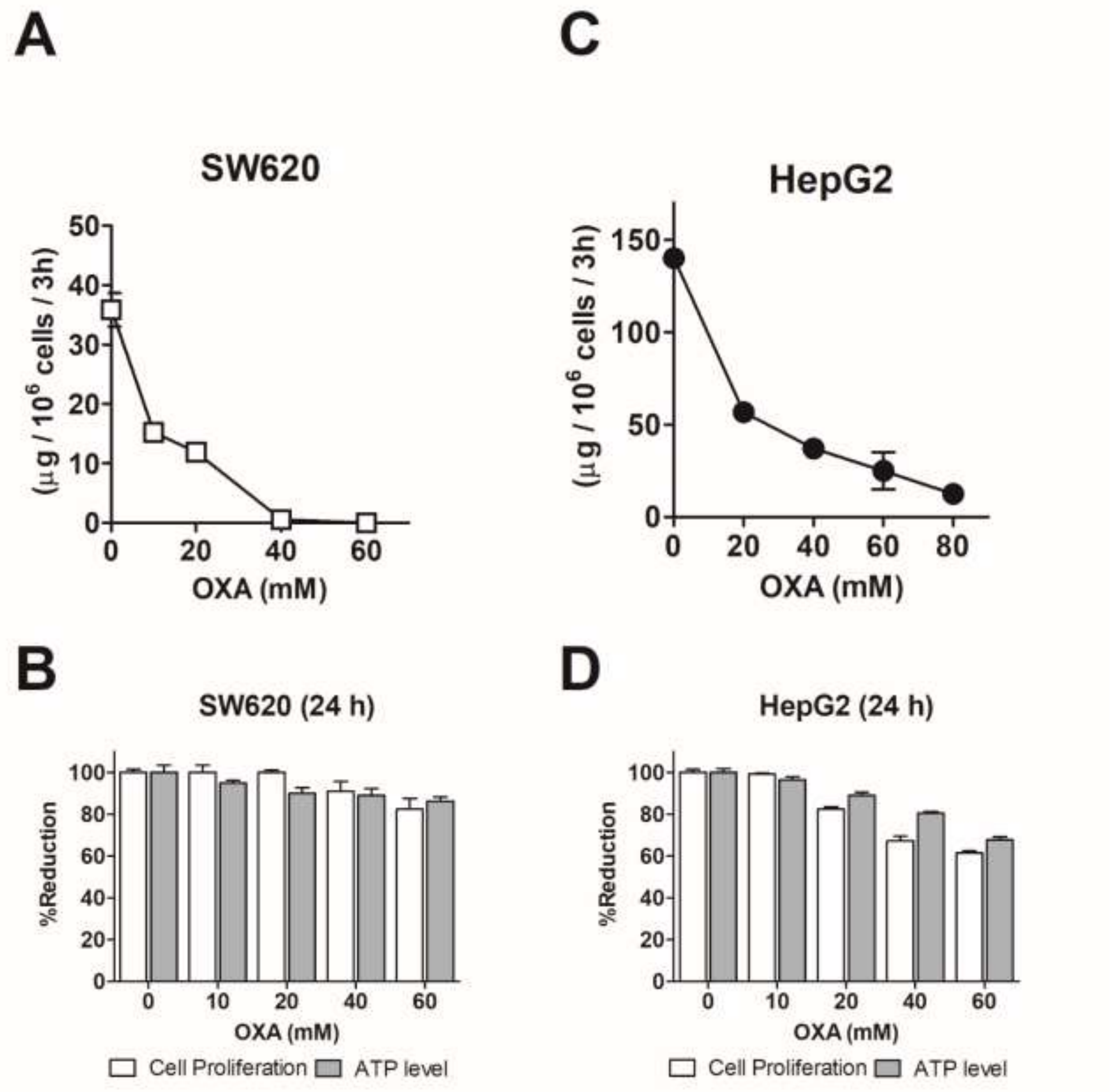

Effects produced by OXA in DMEM-grown SW620 and HepG2 cells. (A) Lactate production, measured in OXA-exposed cells after 3h incubation at 37° in Krebs-Ringer medium. The level of produced metabolite was assessed by using the method described by Farabegoli et al. (Eur J Pharm Sci 2012; 47: 729-738). (B) Cell viability and ATP levels, assessed after a 24 h incubation in the presence of OXA. Cell viability was evaluated using the NR assay (see Materials and Methods). ATP levels were measured by using the CellTiter-Glo Assay (Promega). No statistically significant difference was observed. (C, D) The same experiments were performed on HepG2 cells; (D) a statistically significant effect of cell proliferation and ATP levels was caused by OXA when given at doses >10 mM; p values ranged from <0.05 to <0.01.
